# Supplementary material for: Novel genetically encoded fluorescent probes enable real-time detection of potassium in vitro and in vivo
Source: Nat Commun. 2017 Nov 10;8:1422. doi: 10.1038/s41467-017-01615-z (PMC5681659; doi:10.1038/s41467-017-01615-z)
Supplement: Supplementary file 1 — Supplementary Information [file 41467_2017_1615_MOESM1_ESM.pdf]

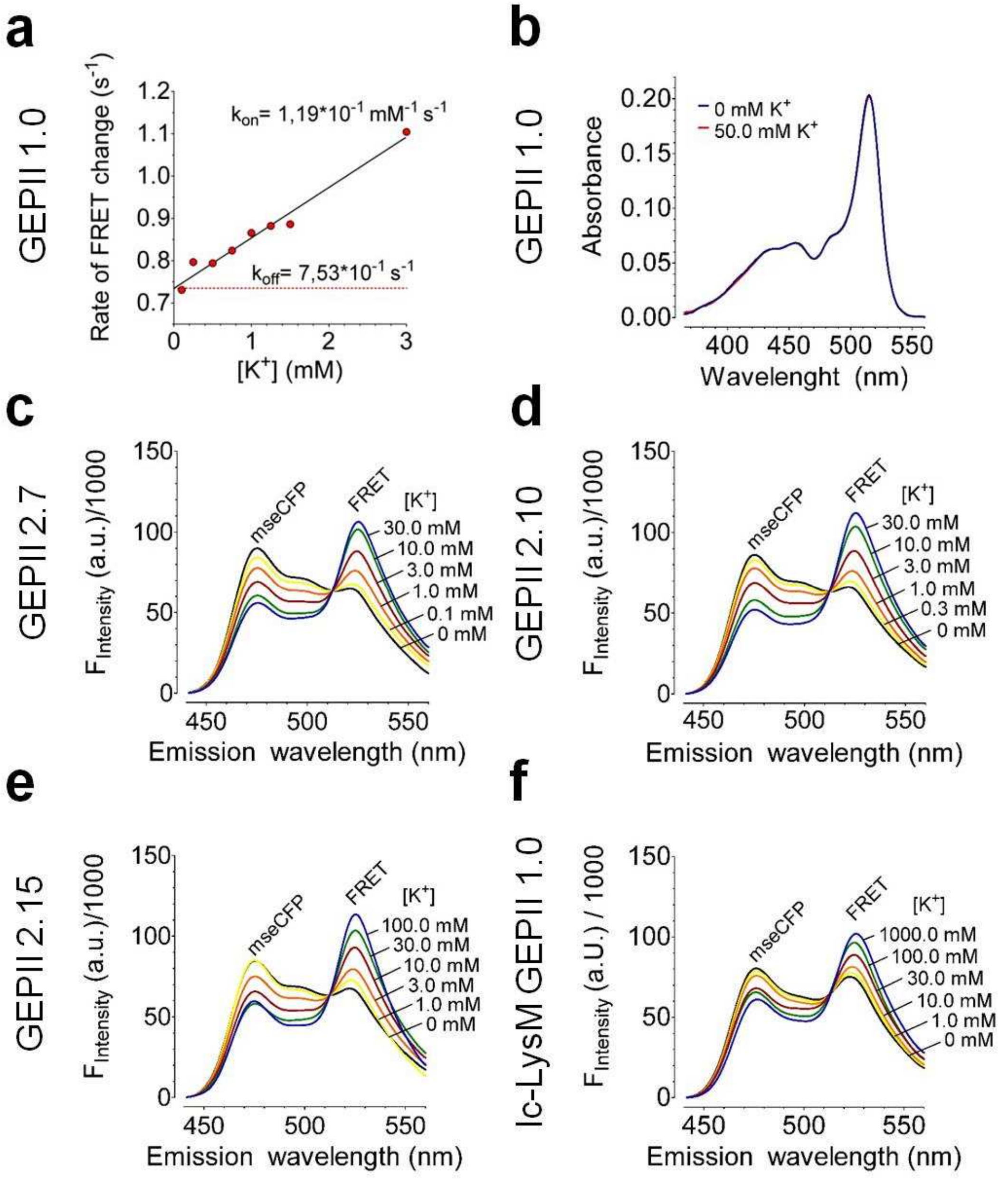

Supplementary Figure 1: Properties of recombinant GEPIIs. (**a**) Reaction rate constants of GEPII 1.0. Apparent rate constants ($k^{app} = k_{on}[K^+] + k_{off}$) at 25 °C, determined by fitting the FRET fluorescence increase after K^+ addition with a single exponential equation, were plotted against K^+ concentrations ($[K^+]$). (**b**) Absorbance spectrum of GEPII 1.0 in the absence (black line) or presence of 50.0 mM K^+ (red line). (**c**) Emission spectra of GEPII 2.7, (**d**) GEPII 2.10, (**e**) GEPII 2.15 and (**f**) lc-LysM GEPII 1.0 at different K^+ concentrations. Purified GEPIIs were imaged in a HEPES-buffered solution (pH=7.3) at room temperature in a 96 well plate using a FRET fluorescence plate reader.

```
GEPII 1.0            MGLFNFVKDAGEKLWDAVTGQHDKDDQAKKVQEHLNKTGIPDADKVNIQIADGKATVTGD
lc-LysM GEPII 1.0    MGLFNFVKDAGEKLWDAVTGQHDKDDQAKKVQEHLNKTGIPDADKVNIQIADGKATVTGD
lc-BON GEPII 1.0     MGLFNFVKDAGEKLWDAVTGQHDKDDQAKKVQEHLNKTGIPNANKVNIQIANGKATVTGN
GEPII 2.7            MGLFNFVKDAGEKLWDAVTGQHDKDDQAKKVQEHLNKTGIPDADKVNIQIADGKATVTGD
GEPII 2.10           MGLFNFVKDAGEKLWDAVTGQHDKDDQAKKVQEHLNKTGIPDADKVNIQIADGKATVTGD
GEPII 2.15           MGLFNFVKDAGEKLWDAVTGQHDKDDQAKKVQEHLNKTGIPDADKVNIQIADGKATVTGD

GEPII 1.0            GLSQEAKEKILVAVGNISGIASVDDQVKTATPATAS---------------QFYTVKSGD
lc-LysM GEPII 1.0    GLSQEAKEKILVAVGNISGIASVDDQVKTATPATAS---------------QFYTVKSGN
lc-BON GEPII 1.0     GLSQQAKQKILVAVGNISGIASVNNQVKTATPATAS---------------QFYTVKSGD
GEPII 2.7            GLSQEAKEKILVAVGNISGIASVDDQVKTATPATASGGGGSGG--------QFYTVKSGD
GEPII 2.10           GLSQEAKEKILVAVGNISGIASVDDQVKTATPATASGGGGSGGGGS-----QFYTVKSGD
GEPII 2.15           GLSQEAKEKILVAVGNISGIASVDDQVKTATPATASGGGGSGGGGSGGGGSQFYTVKSGD

GEPII 1.0            TLSAISKQVYGNANLYNKIFEANKPMLKSPDKIYPGQVLRIPEE
lc-LysM GEPII 1.0    TLSAISKQVYGNANLYNKIFQANKPMLKSPNKIYPGQVLRIPEE
lc-BON GEPII 1.0     TLSAISKQVYGNANLYNKIFEANKPMLKSPDKIYPGQVLRIPEE
GEPII 2.7            TLSAISKQVYGNANLYNKIFEANKPMLKSPDKIYPGQVLRIPEE
GEPII 2.10           TLSAISKQVYGNANLYNKIFEANKPMLKSPDKIYPGQVLRIPEE
GEPII 2.15           TLSAISKQVYGNANLYNKIFEANKPMLKSPDKIYPGQVLRIPEE
```

Supplementary Figure 2: Amino acid sequence alignment of the different Kbp variants used in respective GEPIIs. (**a**) Figure demonstrates the amino acid sequence of GEPII 1.0, lc-LysM GEPII 1.0, lc-BON GEPII 1.0, GEPII 2.7, GEPII 2.10 and GEPII 2.15. Loci of point mutations are indicated in grey, respective exchanged amino acids are highlighted in yellow. Linkers introduced between the BON and LysM domain are highlighted in green, respectively.

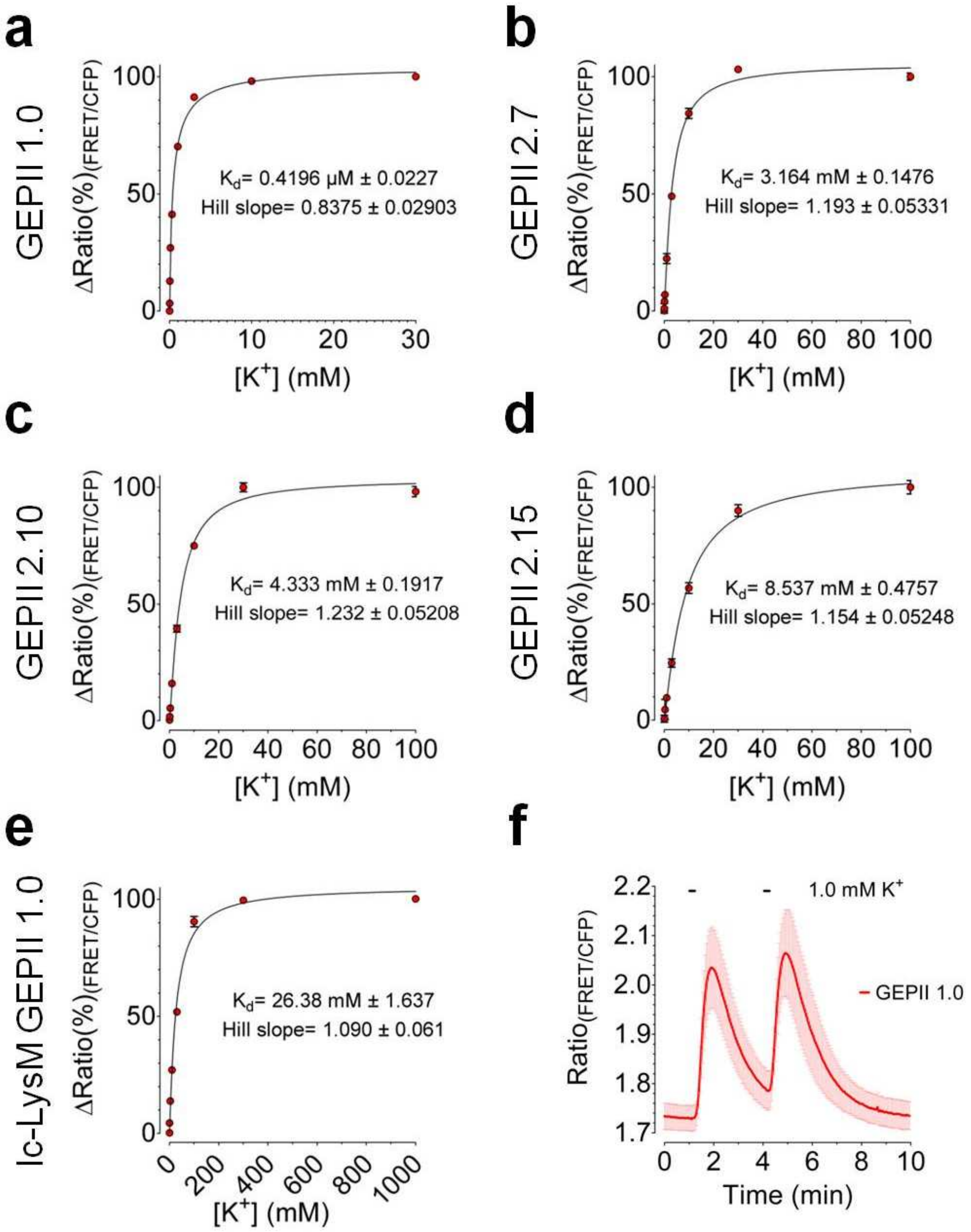

Supplementary Figure 3: K^+ binding properties of GEPIIs. (**a-e**) Panels demonstrate observed responses of GEPIIs upon administration of various K^+ concentrations. Binding kinetics of GEPII 1.0 (**a**), GEPII 2.7 (**b**), GEPII 2.10 (**c**), GEPII 2.15 (**d**) and lc-LysM GEPII 1.0 (**e**) were derived using binding-saturation fit. K_ds and hill slopes are indicated in each panel, respectively. Values represent mean ± SD, n=4 for each. (**f**) GEPII 1.0 embedded in 0.5% agarose was analyzed using a fluorescence microscope in respone to administration and removal of 1.0 mM K^+ via a gravity driven perfusion system. Curve respresents mean ± SD, n=7.

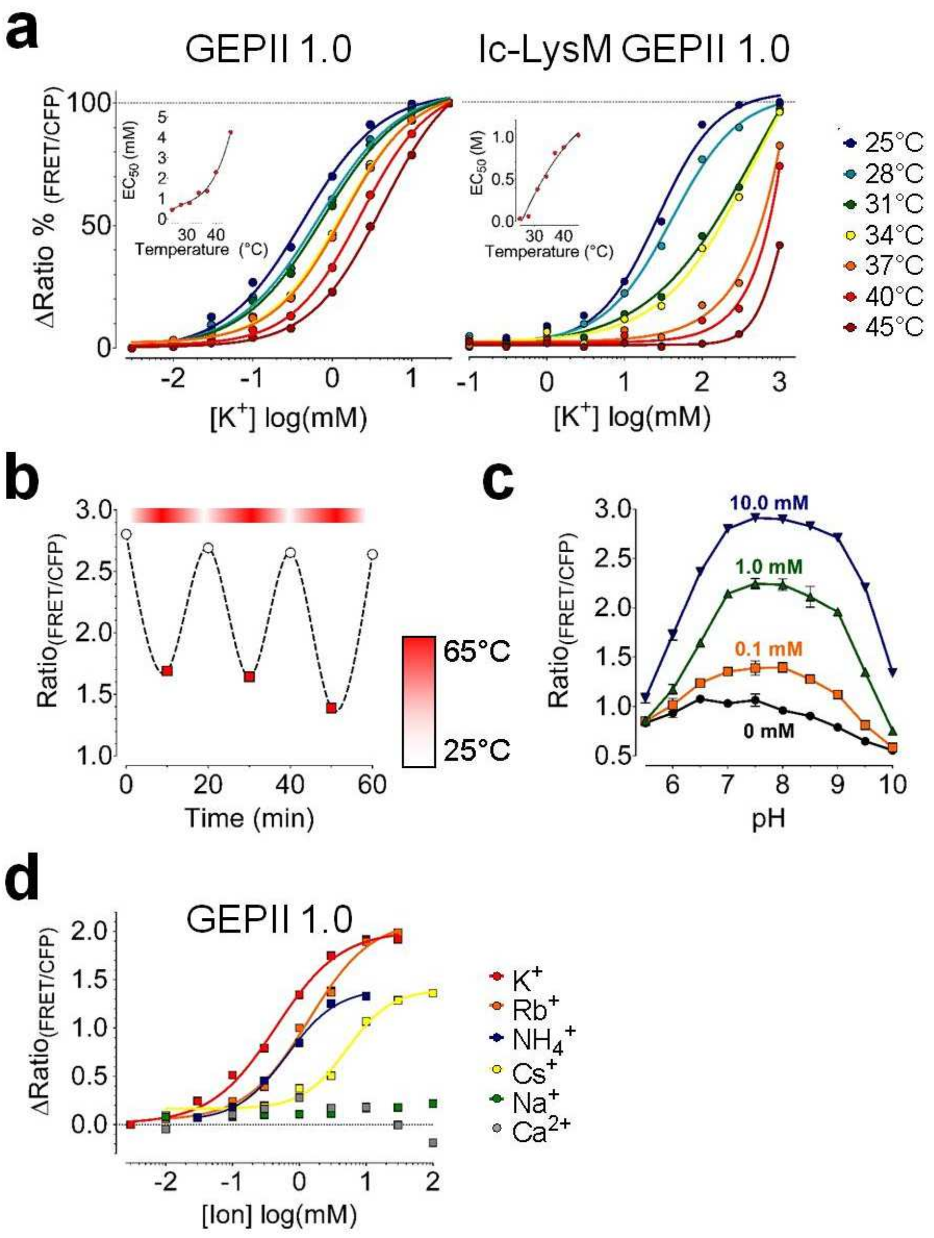

Supplementary Figure 4: Temperature characteristics and specificity of GEPIIs. (**a**) Normalized EC_{50} curves for K^+ of GEPII 1.0 (left panel) and lc-LysM GEPII 1.0 (right panel) at increasing temperatures, ranging from 25°C to 45°C. Determined EC_{50} values of GEPII 1.0 (small left panel) and lc-LysM GEPII 1.0 (small right panel) were plotted over temperature (n=6 for both). (**b**) FRET ratio signal of GEPII 1.0 over time in the presence of 5.0 mM K^+, repetitively recorded at 25°C and 65°C, as indicated. (**c**) pH stability of GEPII 1.0 in the absence or presence of increasing K^+ concentrations (n=8 for each, ± SD). pH shifts were induced using a HEPES-buffered solution by adding HCl or NMDG, respectively. (**d**) K^+ selectivity of GEPII 1.0. FRET ratio signals are plotted against increasing concentrations of different cations (n=3 for each ion, ± SD). Experiments were performed in a 96 well plate using a FRET fluorescence plate reader.

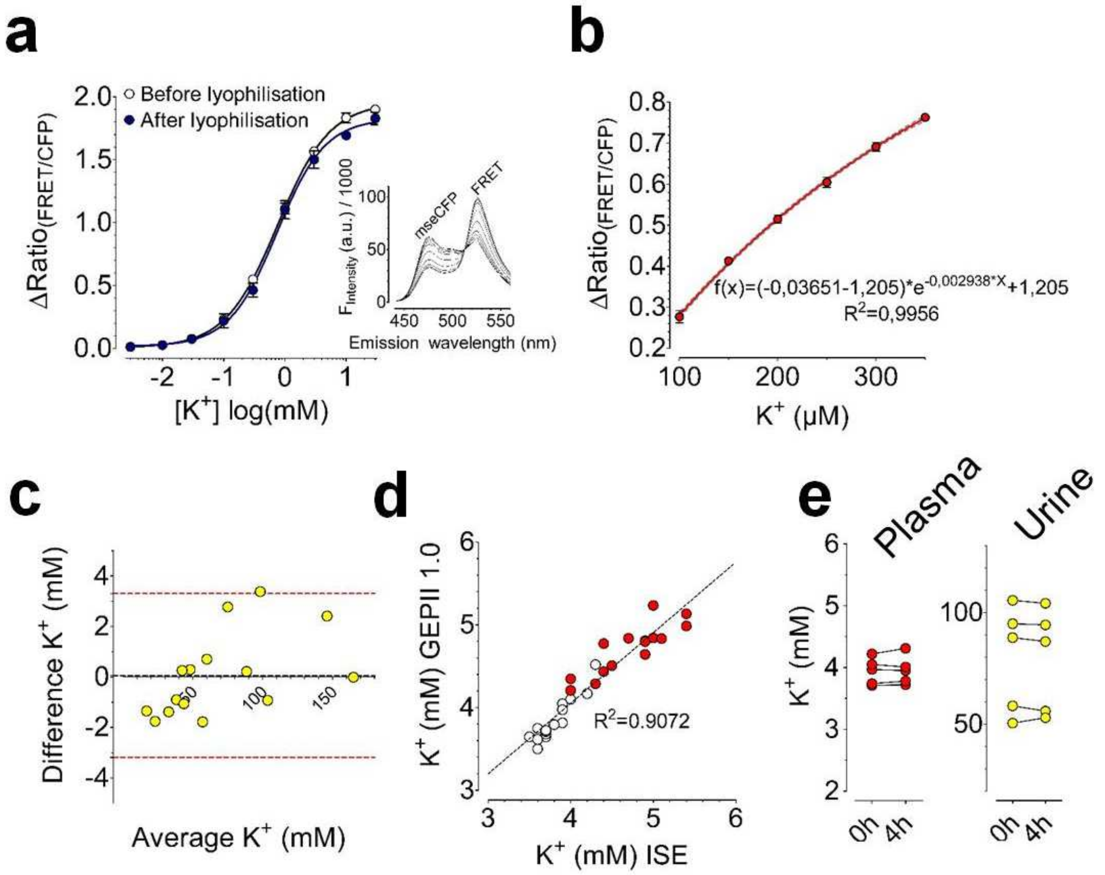

Supplementary Figure 5: Characterization of lyophilized GEPII 1.0 and determination of K^+ concentrations within human plasma and urine samples using purified GEPII 1.0. (**a**) ΔFRET ratio signals of GEPII 1.0 in solution before (white circles) and after lyophilization (blue circles) were plotted against increasing concentrations of K^+ (n=3 for both conditions ± SD). Small panel demonstrates spectral properties of dissolved GEPII 1.0 after lyophilization. (**b**) Calibration curve and -function of recombinant GEPII 1.0 in distilled water using defined KCl concentrations (n=10 ± SD). The curve/function was further used for determinations of K^+ concentrations within biological samples using GEPII 1.0. (**c**) Bland-Altman plot showing urine K^+ levels of healthy human donors. Same samples were separated and used to quantify urine K^+ levels either with GEPII 1.0 or ISE (n=15). Bias (black dashed line) and 95% limits of agreement (red dashed lines) are indicated. (**d**) Plasma K^+ levels of healthy human donors (white circles, n=15) and hemodialysis patients (red circles, n=15) were determined with GEPII 1.0 and plotted against K^+ levels determined with ion selective electrodes (ISE). (**e**) K^+ levels within individual human plasma (left panel) and urine samples (right panel) were determined directly (0h) or 4 hours (4h) after adding GEPII 1.0 to respective samples (n=5 for both, P=0.5238 for plasma and P=0.4444 for urine samples, paired t-test).

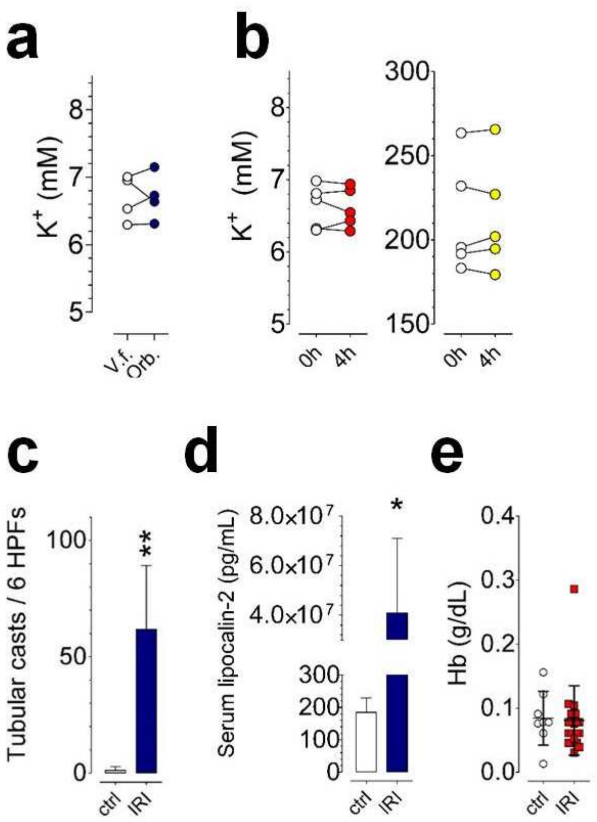

Supplementary Figure 6: Determination of K⁺ levels in murine samples using purified GEPII 1.0. (**a**) Comparison of murine serum K^+ levels determined with recombinant GEPII 1.0. Blood samples were obtained either from the vena facialis (V.f.) or by retro-orbital bleeding (Orb.) using the same mice (n=5, P=0.9319, paired t-test). (**b**) Murine serum (left panel) and urine (right panel) K^+ levels were determined using recombinant GEPII 1.0. Samples were analyzed either directly (0h, white circles) or 4 hours after adding GEPII 1.0 (4h, red and yellow circles) using the same samples (n=5 for both, P=0.7416 for serum, P=0.8134 for urine samples, paired t-test). (**c**) Bars represent tubular casts per 6 high power fields (HPFs) of control mice (ctrl, white bar, n=5 ± SD) and mice after ischemia-reperfusion injury (IRI, blue bar, n=5 ± SD, **P=0.0011, unpaired t-test). (**d**) Serum lipocalin-2 levels of control mice (ctrl, white bar, n=5 ± SD) and mice after IRI (IRI, blue bar, n=5 ± SD). *P=0.0163, unpaired t-test. (**e**) Hemoglobin levels within serum samples of control mice (ctrl, white circles, n= 8 ± SD) and mice after IRI (IRI, red squares, n=19 ± SD, P=0.8725, unpaired t-test). Hemoglobin was determined using Drabkin reagent.

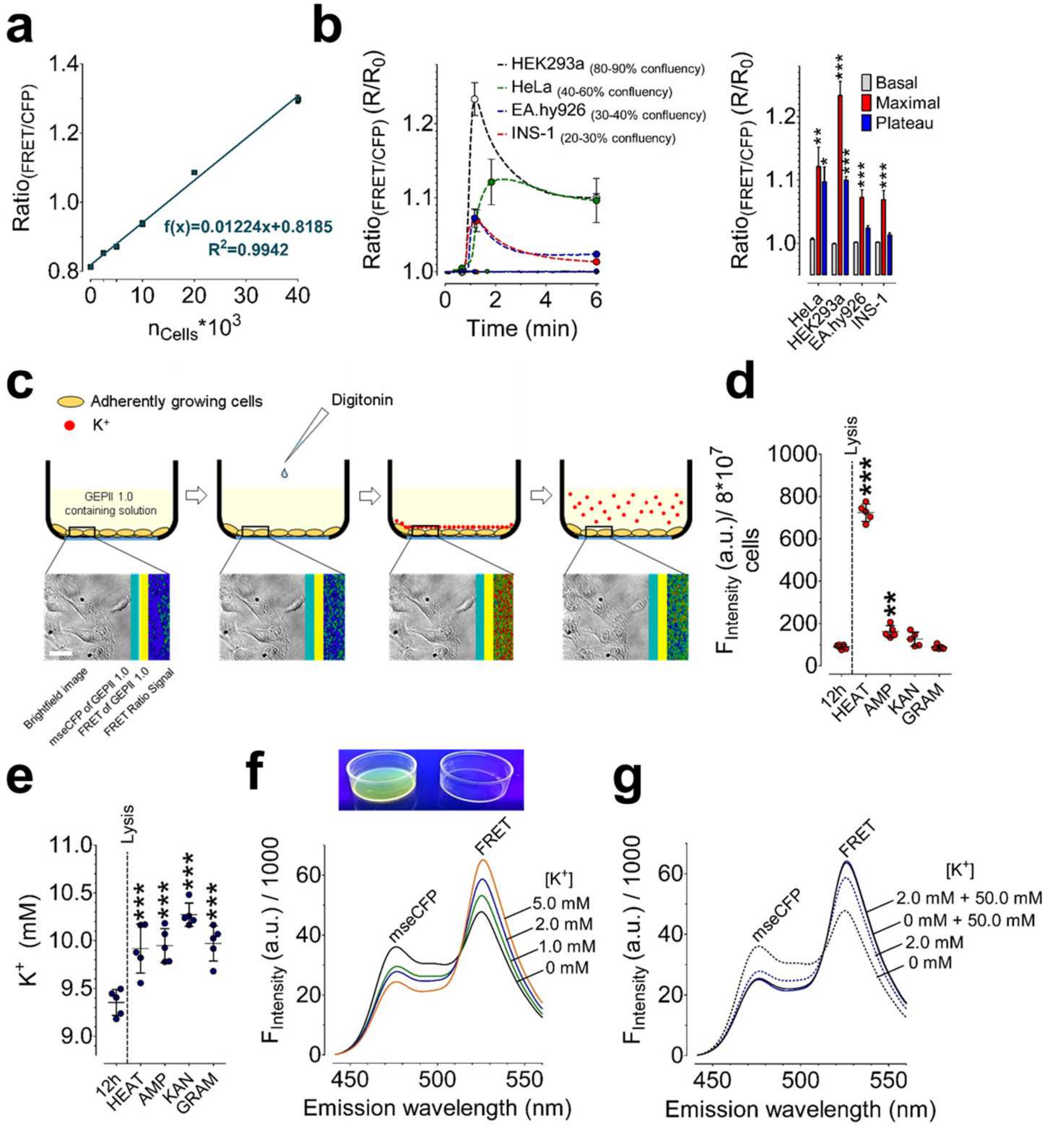

Supplementary Figure 7: $[K^+]_{ex}$ determinations with purified GEPII 1.0 as a dynamic measure of cell viability. (**a**) FRET ratio signals obtained using GEPII 1.0 within the supernatant of digitonin (30 µM) - treated HeLa cells is plotted over respective cell numbers (n=4 ± SD). (**b**) Left panel: normalized FRET ratio signals ± SD over time of purified GEPII 1.0 in the supernatant of adherent EA.hy926 (n=6), HEK293a (n=6), HeLa (n=5) and INS-1 cells (n=6). As indicated, cells of different confluences were treated with 30 µM digitonin (dashed lines) or vehicle (solid lines). Right panel: bars ± SD represent normalized basal FRET ratios (grey bar, basal), maximal ΔFRET ratio signals (red bars), and ΔFRET ratio signals 6 minutes after digitonin addition (blue bar). *P<0.05, **P<0.01, ***P<0.0001, one-way ANOVA test with Tukey's Multiple Comparison Test. (**c**) Schematic representation of co-imaging experiments shown in (**b**). Cell morphology was visualized simultaneously with $[K^+]_{ex}$ using purified GEPII 1.0 in the supernatant of cells by combining wide-field with FRET ratiometric imaging. (**d**) Fluorescence intensities of a propidium iodide (PI) - stained *E.coli* population after 12 hours (12h) of cell growth, heating of the bacteria population for 1 hour at 75°C (HEAT), upon treatment with ampicillin (AMP), kanamycin (KAN) or gramicidin (GRAM) (n=5 for each condition ± SD , **P<0.001, ***P<0.0001, one-way ANOVA test with Tukey's Multiple Comparison Test. (**e**) Bacterial cultures as seen in (**d**) analyzed for $[K^+]_{ex}$ using recombinant GEPII 1.0 (n=5 for each condition ± SD, ***P<0.0001, one-way ANOVA test with Tukey's Multiple Comparison Test). (**f**) Upper panel: petri dishes (35 mm diameter) either filled with agar-agar containing GEPII 1.0 (left dish) or empty (right dish). Lower panel: representative emission spectra of purified GEPII 1.0 in agar-agar containing different K^+ concentrations. (**g**) Emission spectra of agar-agar containing GEPII 1.0 in the absence of K^+ (grey dashed line) or presence of 2.0 mM K^+ in the agar-agar (blue dashed line). As indicated, 50 mM K^+ was added to the GEPII 1.0 containing agar-agar wells which affected the respective emission spectra (solid lines).

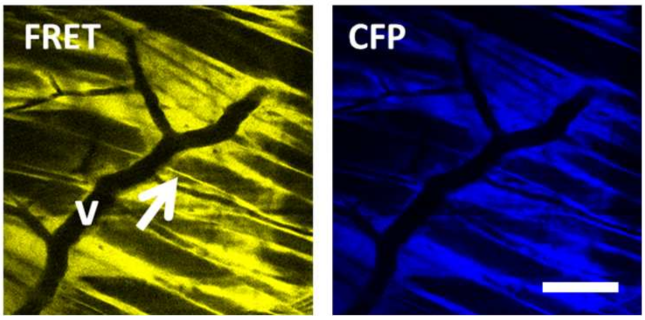

Supplementary Figure 8: Localization of GEPII 1.0 in the cremaster muscle *in vivo.* Single representative z-plain images at 50 µm depth of FRET (left image) and mseCFP (right image) of recombinant GEPII 1.0 loaded in the mouse cremaster muscle. Blood vessels (v) and localization of GEPII 1.0 along muscle fibers (arrow) are indicated. Scale bar represents 100 µm.

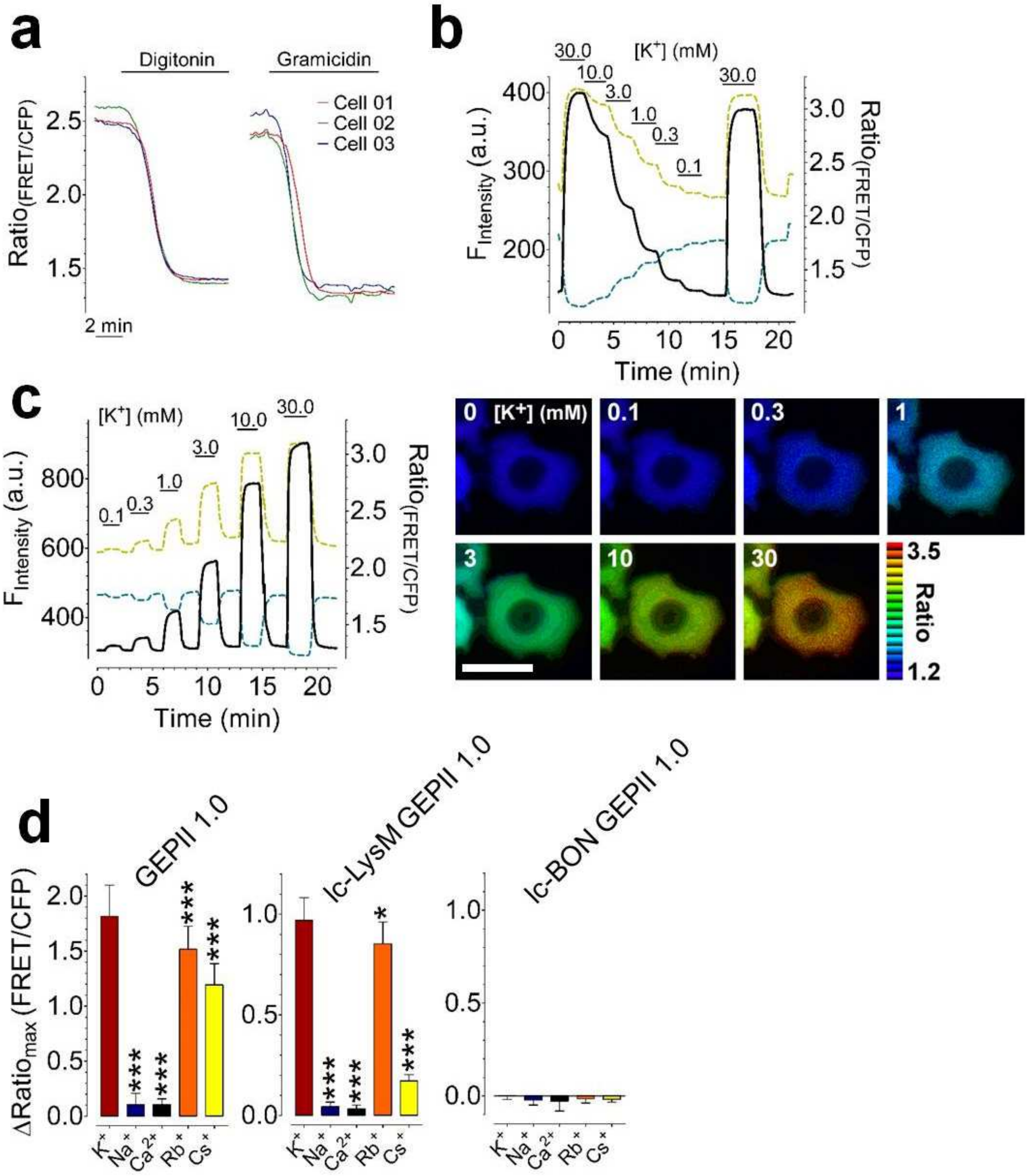

Supplementary Figure 9: Characterization of GEPIIs expressed in single living cells. (**a**) Representative single cell responses of HeLa cells expressing lc-LysM GEPII 1.0 treated either with 5 µM digitonin (left panel, n=4) or 10 µM gramicidin (right panel, n=4) at times indicated. (**b**) Representative response of a HeLa cell expressing GEPII 1.0, treated with 5 µM digitonin. Fluorescence intensities of FRET (yellow dashed line) and mseCFP (cyan dashed line) were recorded upon stepwise decreasing extracellular K^+ concentration from 30.0 mM to 0 mM. The FRET ratio signal (black solid line) is shown over time in the same diagram. (**c**) Fluorescence intensities (left panel) of FRET (yellow dashed line) and mseCFP (cyan dashed line) of a permeabilized HeLa cell expressing GEPII 1.0 treated with 5 µM digitonin were recorded upon stepwise increasing and removing $[K^+]_{ex}$ (left panel). The FRET ratio signal over time (black solid line, left panel) and representative pseudocolored FRET ratio images (right images) are shown. Scale bar in the lower left image represents 20 µm. (**d**) Maximal ΔFRET ratio signals ± SD of permeabilized HeLa cells expressing GEPII 1.0 (left panel), lc-LysM GEPII 1.0 (middle panel) or lc-BON GEPII 1.0 (right panel) upon addition of 3 mM K^+, Na^+, Ca^{2+}, Rb^+, or Cs^+ for GEPII 1.0 (left panel, n=4 for each ion), or 140 mM K^+, Na^+, Rb^+ and Cs^+, or 90 mM Ca^{2+} for lc-LysM GEPII 1.0 (middle panel, n=3 for each ion) and lc-BON GEPII 1.0 (right panel, n=4 for each ion). *P<0.05, ***P<0.0001, one-way ANOVA test with Tukey's Multiple Comparison Test).

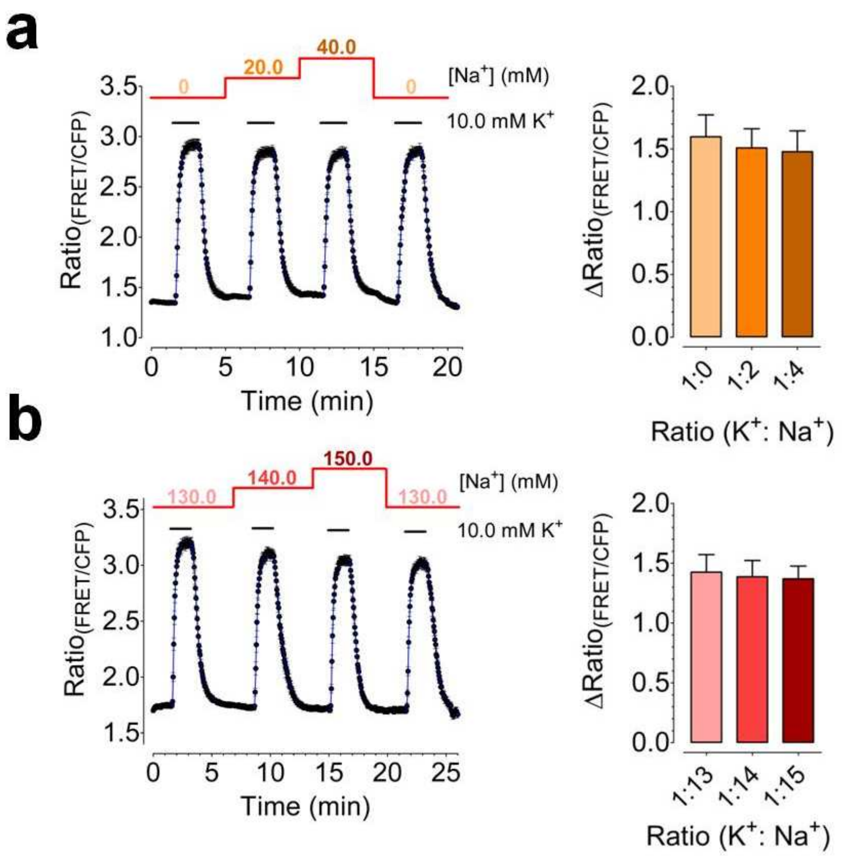

Supplementary Figure 10: Effects of Na^+ on K^+ sensing with GEPII 1.0. HeLa cells expressing GEPII 1.0 were treated with 5 µM digitonin, 10 µM monensin and 5 µM valinomycin for permeabilization. (**a**) FRET ratio signal over time of GEPII 1.0 upon administration of 10.0 mM K^+ was recorded in the absence or presence of 20 .0 or 40.0 mM Na^+ (left panel). Maximal received ΔRatio signals are demonstrated (right panel). n=4, one-way ANOVA test with Tukey's Multiple Comparison Test. (**b**) FRET ratio signal over time of GEPII 1.0 upon administration of 10.0 mM K^+ was recorded in the presence of 130.0, 140.0 or 150.0 mM Na^+ (left panel). Maximal received ΔRatio signals did are demonstrated (right panel). n=3, one-way ANOVA test with Tukey's Multiple Comparison Test.

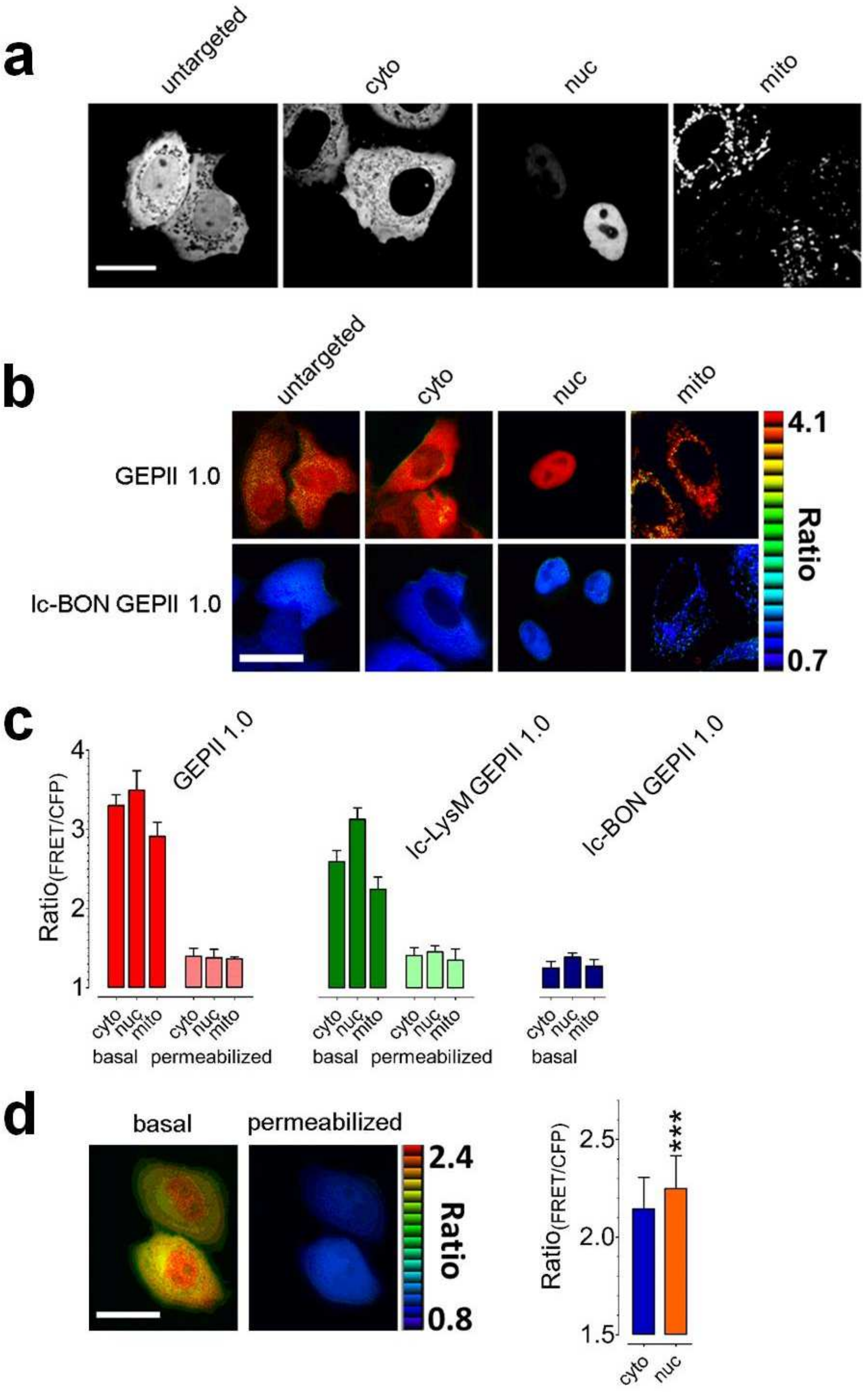

Supplementary Figure 11: Targeting of GEPIIs to subcellular organelles. (**a**) Representative confocal microscopy images of HeLa cells expressing either untargeted (left image), cytosolic (middle left image), nuclear (middle right image) or mitochondrial (right image) targeted GEPII 1.0. Scale bar represents 20 µm. (**b**) Representative pseudocolored FRET ratio images of HeLa cells expressing either GEPII 1.0 (upper images) or the K^+-insensitive lc-BON GEPII 1.0 (lower images). GEPIIs were either untargeted (left images), targeted to the cytosol (middle left images), the mitochondria (middle right images) or the nucleus (right images). Scale bar in the lower left image represents 20 µm. (**c**) Basal FRET ratio values ± SD of GEPII 1.0 (left panel, n=3), lc-LysM GEPII 1.0 (middle panel, n=6) or lc-BON GEPII 1.0 (right panel, n=5) under basal conditions of intact cells (first three columns, all panels) or after cell permeabilization using 5 µM of digitonin (right three columns, left and middle panel). (**d**) Representative pseudo-colored ratio images of HeLa cells expressing untargeted lc-LysM GEPII 1.0 under basal condition of intact cells (left image) and same cells after cell permeabilization using 5 µM of digitonin (right image). Scale bar represents 20 µm. Bars in the right panel represent FRET ratio signals of cytosolic (blue bar) and nuclear (orange bar) regions of same cells expressing untargeted lc-LysM GEPII 1.0 (n=5 ± SD , ***P<0.0001, paired t-test).

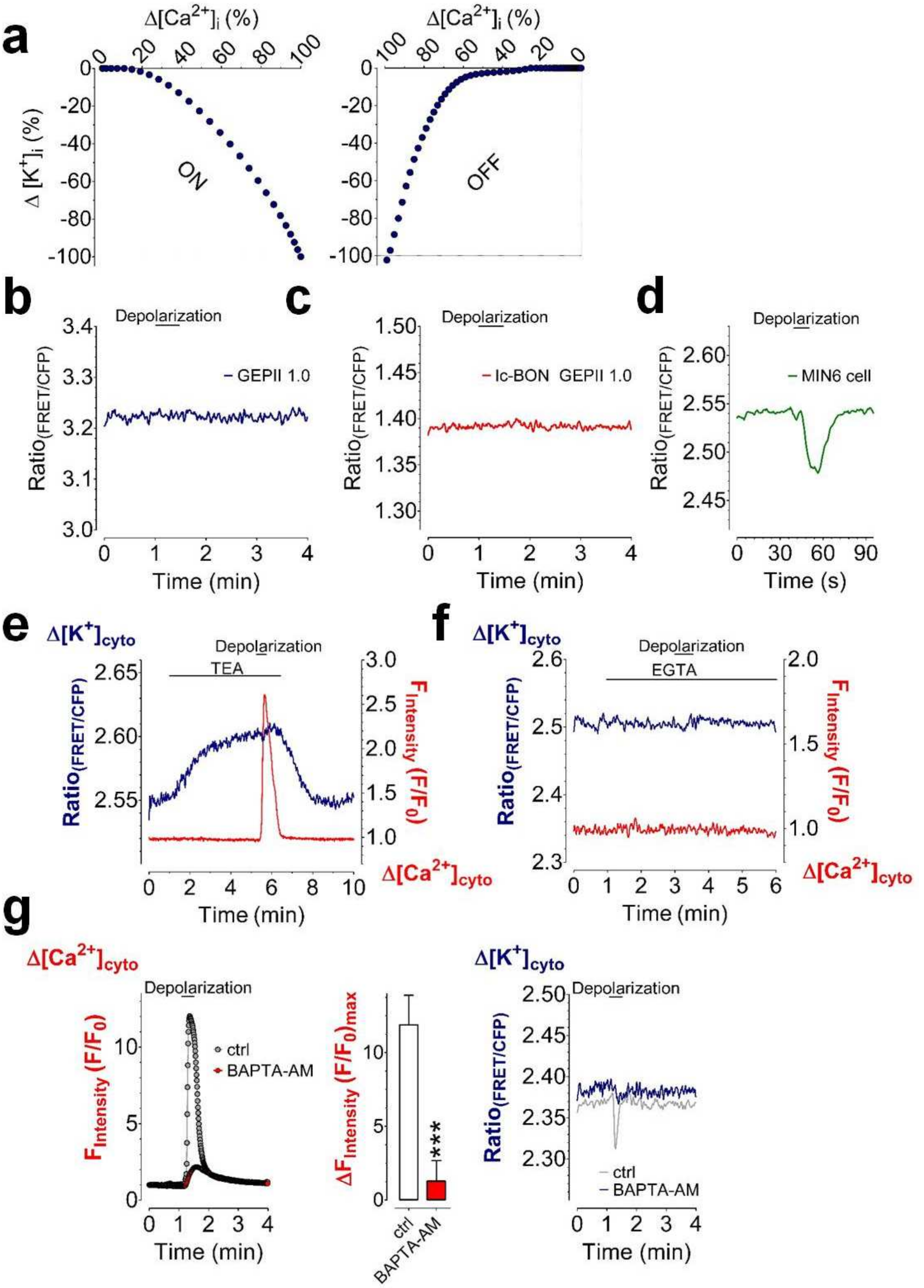

Supplementary Figure 12: Characterization of Ca^{2+}-induced K^+ fluxes within INS-1 cells. (**a**) Representative on (left panel) and off (right panel) kinetics of the Ca^{2+}-induced K^+ transient within an INS-1 cell. $\Delta[K^+]_i$(%) is plotted over $\Delta[Ca^{2+}]_i$(%). (**b**) Representative single cell response of an INS-1 cell expressing GEPII 1.0 upon cell depolarization using 70 mM KCl (n=6 independent measurements/ 43 cells were analyzed/ 43 cells responded as demonstrated). (**c**) Representative single cell response of an INS-1 cell expressing the K^+-insensitive lc-BON GEPII 1.0 upon cell depolarization with 70 mM KCl (n=8/ 40/ 40). (**d**) Representative single cell response of a MIN6 cell expressing lc-LysM GEPII 1.0 in response to depolarization using 70 mM KCl (n=8/ 24/ 24). (**e**) Demonstrative single cell response of an INS-1 cell expressing lc-LysM GEPII 1.0 (K^+, blue curve) and CAR-GECO1 (Ca^{2+}, red curve) upon cell depolarization in the presence of 15 mM TEA (n=3/ 28/ 19). (**f**) Representative single cell response of an INS-1 cell simultaneously expressing lc-LysM GEPII 1.0 (K^+, blue curve) and CAR-GECO1 (Ca^{2+}, red curve) upon depolarization in the absence of extracellular Ca^{2+} (n=7/ 57/ 57). (**g**) Average responses of INS-1 cells expressing CAR-GECO1 (left panel) upon cell depolarization under control conditions (grey circles, n=3/ 19) or cells loaded with BAPTA-AM (red squares, n=5/ 56) and corresponding maximal Δ fluorescence intensities (middle panel) under same conditions (ctrl, white bar, n=3/ 19 and BAPTA-AM, red bar, n=5/ 56, ***P<0.0001, unpaired t-test). K^+ signals are demonstrated as representative single cell responses (right panel). INS-1 cells expressing lc-LysM GEPII 1.0 were depolarized with 70 mM KCL under control conditions (right panel, grey curve, n=3/ 20/ 20) or after cell loading with BAPTA-AM (right panel, blue curve, n=6/ 53/ 50).

Supplementary Table 1: Sensitivity and specificity parameters of GEPII 1.0 variants

GEPII variant	*Limit of detection [K^+] (mM)*	*EC_{50} [K^+] (mM) i.v./ i.s.*	*Hill Slope (K^+) i.v./ i.s.*	*EC_{50} [Na^+] (mM) i.v.*	*EC_{50} [Rb^+] (mM) i.v.*	*EC_{50} [Cs^+] (mM) i.v.*	*EC_{50} [NH_4^+] (mM) i.v.*	*EC_{50} [Ca^{2+}] (mM) i.v.*
GEPII 1.0	≤ 0.01	0.42/ 0.82	0.83/ 1.15	-	1.38	5.06	1.94	-
lc-LysM GEPII 1.0	≤ 1.0	30.47/ 60.95	1.17/ 1.22	-	42.58	-	-	-
lc-BON GEPII 1.0	-	-	-	-	-	-	-	-

Supplementary Table 2: Sensitivity parameters of GEPII 2.7, 2.10, and 2.15

GEPII variant	*Linker length (amino acids)*	*Amino acid composition of linker*	*Limit of detection [K^+] (mM)*	*EC_{50} [K^+] (mM) i.v./ i.s*	*Hill Slope i.v./ i.s.*
GEPII 2.7	7	GGGGSGG	≤ 0.1	3.24/ 9.09	1.25/ 1.14
GEPII 2.10	10	GGGGSGGGGS	≤ 0.1	4.39/ 10.11	1.27/ 1.26
GEPII 2.15	15	GGGGSGGGGSGGGGS	≤ 0.1	8.59/ 15.59	1.20/ 1.16

Supplementary Table 3: Primers for the generation and targeting of GEPIIs

Primer Name	***Primer Sequence (5'-3')***
Kbp for	GGCATCGATATGGGTCTGTTCAATTTTGTG
Kbp rev	GCTGAATTCCTCTTCCGGAATACGCAAC
lc-BON 01 fwd	CCTGAGTCAGCAGGCGAAGAACAAAATCCTTGTTGCGGTGG GGAATATTTCCGGTATTGCCAGTGTCAATAATCAGGTG
lc-BON 02 fwd	CCAATAAAGTGAATATTCAAATTGCCAACGGCAAAGCGACGGTCACTGGTAACGGCCTG AGTCAGCAGGCGAAGAAC
lc-BON 03 fwd	AGTAAGAAGGTGCAGCAGCATCTGAACAAAACCGGTATACCGAATGCCAATAAAGTGA ATATTC
lc-BON 04 fwd	TCTGTTCAATTTTGTGAAAGATGCCGGAGAAAAACTCTGGGACGCGGTTACAGGTCAGC ACGATAAAGACGATCAGGCGAAGAAGGTGCAGCAGCATCTG
lc-BON 05 fwd	GGCATCGATATGGGTCTGTTCAATTTTGTG
lc-LysM 01 fwd	TTTTTAGCATCGGTTTATTCGCTTGGAAGATTTTATTGTACAGATTAGCGTTACCGTAGAC CTGTTTGGAAATGGCACTCAGAGTGTTGCCAGACTTAAC
lc-LysM 02 fwd	TGAGAATTCCTCTTCCGGAATACGCAACACTTGCCCCGGATAAATTTTATTCGGGCTTTTT AGCATCGGTTTATTCGCTTG
GEPII 2.7 fwd	GGCGGAGGCGGAAGCGGCGGACAGTTTTATACCGTTAAGTCTGGC
GEPII 2.7 rev	TCCGCCGCTTCCGCCTCCGCCGCTGGCAGTGGCTGGTGTCGCCG
GEPII 2.10 fwd	GGCGGAGGCGGAAGCGGCGGAGGCGGATCCCAGTTTTATACCGTTAAGTCTGGC
GEPII 2.10 rev	GGATCCGCCTCCGCCGCTTCCGCCTCCGCCGCTGGCAGTGGCTGGTGTCGCCG
GEPII 2.15 fwd	GGCGGAGGCGGAAGCGGCGGAGGCGGATCCGGCGGAGGCGGAAGCCAGTTTTATACC GTTAAGTCTGGC
GEPII 2.15 rev	GCTTCCGCCTCCGCCGGATCCGCCTCCGCCGCTTCCGCCTCCGCCGCTGGCAGTGGCTG GTGTCGCCG
cpV fwd	GAATTCATGGACGGCGGCGTG
NLS cpV rev	AGCAAGCTTAGCAAAACGCCATGCTCCAGCTGCGTTT GGTACCCTCGATGTTGTGGCGGATCTTGAAG
NES cpV rev	CCTAAGCTTATAACGTCAATCGTTCTAATGGAGGCAAGGTACCCTCGATGTTGTGGCGG ATCTTGAAG
CAAX cpV rev	AAGCTTTTACATAATTACACACTTTGTCTTTGACTTCTTTTTCTTCTTTTTAACATCTTTGCT CATGGTACCCTCGATGTTGTGGCGGATCTTGAAG
