# Supplementary material for: Novel genetically encoded fluorescent probes enable real-time detection of potassium in vitro and in vivo
Source: Nat Commun. 2017 Nov 10;8:1422. doi: 10.1038/s41467-017-01615-z (PMC5681659; doi:10.1038/s41467-017-01615-z)
Supplement: Supplementary file 3 — Description of Additional Supplementary Files [file 41467_2017_1615_MOESM3_ESM.pdf]

Description of Additional Supplementary Files

File Name: Supplementary Movie 1
Description: Imagining of cellular K+ release in response to a necrotic stimulus using purified GEPII 1.0 in the supernatant of HeLa cells. Left panel represents pseudo-colored extracellular FRET ratio signals over time of purified GEPII 1.0 (5 µM), which was added to the external medium of attached HeLa cells. Right panel represents phase contrast images of same HeLa cells. As indicated 30 µM digitonin was added to stimulate cellular K+ release and necrotic cell death.

File Name: Supplementary Movie 2
Description: Imagining of cellular K+ release in response to a necrotic stimulus using purified GEPII 1.0 in the supernatant of different cell types. Pseudo-colored images represent FRET ratio changes of purified GEPII 1.0 (5 µM), which was added to the external medium of INS-1, HeLa, HEK293a, and Ea.hy926 cells. Simultaneously to FRET ratio imaging, phase contrast images were recorded. As indicated cells were treated with 30 µM digitonin.
